# The Sensitivity and Specificity of Potassium Hydroxide Smear and Fungal Culture Relative to Clinical Assessment in the Evaluation of Tinea Pedis: A Pooled Analysis

**DOI:** 10.1155/2010/764843

**Published:** 2010-06-22

**Authors:** Jacob Oren Levitt, Barrie H. Levitt, Arash Akhavan, Howard Yanofsky

**Affiliations:** ^1^Department of Dermatology, The Mount Sinai School of Medicine, New York, NY 10029, USA; ^2^Taro Pharmaceuticals U.S.A., Inc. Hawthorne, NY 10532, USA; ^3^Department of Medicine, McGill University, Montreal, QC, Canada H3G 1Y6

## Abstract

*Background*. There are relatively few studies published examining the sensitivity and specificity of potassium hydroxide (KOH) smear and fungal culture examination of tinea pedis. *Objective*. To evaluate the sensitivity and specificity of KOH smear and fungal culture for diagnosing tinea pedis. *Methods*. A pooled analysis of data from five similarly conducted bioequivalence trials for antifungal drugs was performed. Data from 460 patients enrolled in the vehicle arms of these studies with clinical diagnosis of tinea pedis supported by positive fungal culture were analyzed 6 weeks after initiation of the study to determine the sensitivity and specificity of KOH smear and fungal culture. *Results*. Using clinical assessment as the gold standard, the sensitivities for KOH smear and culture were 73.3% (95% CI: 66.3 to 79.5%) and 41.7% (34.6 to 49.1%), respectively. The respective specificities for culture and KOH smear were 77.7% (72.2 to 82.5%) and 42.5% (36.6 to 48.6%). *Conclusion*. KOH smear and fungal culture are complementary diagnostic tests for tinea pedis, with the former being the more sensitive test of the two, and the latter being more specific.

## 1. Introduction

Tinea pedis is a dermatophyte infection of the plantar feet or toe web spaces most commonly caused by *Trichophyton rubrum*, *Trichophyton mentagrophytes, *and *Epidermophyton floccosum *[1]. It is believed that up to 70% of the world's population will be affected by tinea pedis at some point in their lives [2]. 

Although often easily diagnosed by clinical examination, confirmation of tinea pedis infection by diagnostic tests helps in differentiating the disease from other skin conditions that produce a similar clinical picture [3]. Two commonly used laboratory methods for the diagnosis of tinea pedis are fungal culture and potassium hydroxide (KOH) examination of skin scrapings for fungal elements. While we often rely on KOH smear and/or culture in the diagnosis of tinea pedis, the sensitivity and specificity of these procedures have only been reported in a limited number of studies. In 1993, Miller and Hodgson reported a sensitivity of 77% of KOH examination using culture as a gold standard (i.e., of 139 positive cultures, 107 were positive by KOH examination). Interestingly, of 194 culture-negative specimens, 74 were positive by KOH examination, yielding a specificity of 62% [4]. Of these, one must assume that some of these positive KOH examinations were true positives in spite of negative culture. That is, the discordance between fungal culture and KOH examination results is too great. Therefore, one must question the validity of using culture as a gold standard. 

In one recent study of 2,427 patients, the sensitivity and specificity of KOH examination of tinea pedis were found to be 95.7% and 69.6%, respectively, relative to a culture gold standard [5]. Interestingly, the study found that the percentage of patients presenting with a clinical diagnosis of tinea pedis that had skin cultures positive for fungus were only 36.6%, raising a question as to whether culture is the optimal gold standard by which to evaluate diagnostic tests for tinea pedis. At least one other study showed similar incongruence between the clinical diagnosis of tinea pedis and fungal culture, with less than a third of patients presenting with a clinical diagnosis of the disease having positive cultures [3]. Examples of how culture may miss a diagnosis of tinea pedis include sampling error from the affected foot, using defective culture medium, and mishandling of the culture medium.

Establishing a gold standard for a test is sometimes straightforward but sometimes difficult, as is the case for tinea pedis. For lung cancer, for example, one might compare chest X-ray relative to a gold standard of tissue histology. Here, there is little question that tissue histology nearly uniformly makes or breaks the diagnosis whereas chest X-ray may leave you guessing. And, indeed, one naturally assigns specificity and sensitivity to the test relative to a gold standard, which axiomatically must have essentially 100% sensitivity and 100% specificity. With fungal culture, one does not always culture out positive organisms in the face of a true infection, as defined by one possible gold standard of positive KOH smear and clinical evidence of infection. Indeed, a second sampling at the same or later date may reveal a positive culture even though the initial sample gave a negative result. While some may wish to define fungal culture as the axiomatic gold standard for KOH exam (because you can see and speciate the exact organism), it is not the same caliber of gold standard as, say, tumor histology might be for a chest X-ray in lung cancer.

The dilemma of a lack of gold standard for tinea pedis diagnosis thus poses a problem. In the clinic, we accept positivity of culture or KOH smear as indicative of infection because, even in the event of a false positive, harm from topical treatment is nil. Negative tests may not deter therapy on the basis of clinical suspicion since the tests are imperfect, and again, risks of topical therapy are nil. If the disease did not improve after a 1-week trial of topical antifungals, other diagnoses, such as plantar psoriasis, might be entertained and treated with, say, a topical steroid. What is occurring is that treatment is being chosen on the basis of a positive clinical diagnosis, albeit that culture and KOH smears may be supportive of that decision when positive. That is, clinical diagnosis—albeit imperfect and subjective—is the gold standard in practice.

For clinical trials, such as those used to prove efficacy at the U.S. Food and Drug Administration (FDA), the choice of gold standard by which to define infection would ideally be perfect and objective—a luxury we do not have with one metric at one point in time. In this case, the concern is not in one test predicting a true outcome but rather in the outcome itself. Therefore, for FDA studies, one defines positive as a triple positive (clinical, KOH, and culture) and negative as a triple negative [6]. 

In this pooled analysis of data from five similarly conducted bioequivalence trials for antifungal drugs, we seek to establish the sensitivity and specificity of KOH and culture in the diagnosis of tinea pedis, using clinical assessment as the gold standard.

## 2. Methods

### 2.1. Study Population and Endpoints

The 484 patients in this pooled analysis were participants, randomized to vehicle, in five previous 3-way, randomized, parallel group, double-blind, vehicle-controlled clinical bioequivalence trials involving the use of test and reference antifungal agents against tinea pedis infection. Institutional review board approval was obtained for each of the five studies. The earliest study (STUDY #1), Nizoral (ketoconazole) cream 2% versus ketoconazole cream 2%, was conducted in 2001 and involved 292 patients (192 active and 100 vehicle). The second study (STUDY #2), Spectazole (econazole nitrate) cream 1% versus econazole nitrate cream 1%, was conducted in early 2002 and involved 252 patients (165 active and 87 vehicle). The third study (STUDY #3), Lotrisone (clotrimazole 1%/betamethasone dipropionate 0.05%) lotion versus clotrimazole 1%/betamethasone dipropionate 0.05% lotion, was conducted in late 2002 and involved 399 patients (312 active and 87 vehicle). The fourth study (STUDY #4), Loprox (ciclopirox olamine) cream 0.77% versus ciclopirox olamine cream 0.77%, was conducted in early 2003 and involved 462 patients (373 active and 89 vehicle). The latest study (STUDY #5), Loprox (ciclopirox olamine) topical suspension 0.77% versus ciclopirox olamine topical suspension 0.77%, was conducted in late 2003 and involved 603 patients (482 active and 121 vehicle). All of the studies selected for this pooled analysis were similar in terms of study design, patient characteristics, inclusion and exclusion criteria, disease entity investigated, treatment regimens, and outcome variables measured. 

All participants in these studies were either healthy males or nonpregnant, nonnursing females, aged 18 and over. At study onset, the participants were diagnosed clinically by one of the authors (HY) with tinea pedis, defined by an erythema score of at least 1/3, a pruritus score of at least 1/3, a scaling score of at least 2/3, and a total score of at least 4/18. The participants also had a positive KOH smear and had a positive culture for *Trichophyton rubrum*, *Trichophyton mentagrophytes*, or *Epidermophyton floccosum* prior to randomization. There were 1,524 patients randomized to receive the active treatments and 484 to receive the vehicles.

The subpopulations of interest in this pooled analysis were patients randomized to the vehicle arms in the above-mentioned therapeutic equivalence trials. Fungal culture (positive/negative), KOH smear (positive/negative), and clinical efficacy (presence/absence of disease) assessments were made 4 and 6 weeks after initiation of a 4-week treatment regimen. Clinical assessment was made for a target area of one foot using each of six clinical parameters—erythema, scaling, fissuring, bulla formation, itching, and burning—evaluated on a 0–3 scale, where 0 = none, 1 = mild, 2 = moderate, and 3 = severe. Clinical cure was achieved if severity scores for each of the six parameters were 0 or 1 with a combined score of 2 or less. That said, for the purposes of this manuscript, it is not cure per se that is of interest, but rather, clinical assessment (regardless of outcome) as it compares to KOH and culture outcomes.

The vehicle arms of the studies, rather than both vehicle and active arms, were chosen simply to enrich the proportion of positive KOH or culture readings at the end of the study; however, on theoretical grounds, we could have used all patients. The vehicles were not identical between studies, but they need not be. The current analysis is concerned only with the correlation of KOH or culture to a separate, gold standard assessment, regardless of what cream was used in the area being evaluated. Sensitivity and specificity are inherent properties of a test. It does not matter how the disease cleared (i.e., what creams, if any, were used) but rather, how well the test (KOH or culture) reflects the actual condition (the gold standard). Also, the assessment is of a specific area of the foot from which material for KOH and culture was taken. Clinical assessment of the area in question, rather than of the whole foot, is of interest because we are examining KOH and culture of material taken from that specific area. 

The requirement of culture, KOH, and clinically positive patients at baseline was also theoretically extraneous; however, it served to decrease the number of clinical false positives due to nondermatophyte conditions. In reality, sensitivity and specificity, and the derivative values of PPV and NPV, are calculated from an assessment at one point in time. At that time, true positives and true negatives are defined by the choice of gold standard, in this case clinical assessment. In this vein, we arbitrarily chose six weeks as the time point to compare all the studies. In theory, we could have used the four-week data alone or combined the four-and six-week data. It was felt that the pooled six-week data provided a sufficient sample size to draw conclusions, which are at best a range of values anyway.

Finally, we opted not to analyze the combination of KOH smear *and* culture relative to clinical assessment. The permutations of double positives, double negatives, or discordant pairs of KOH smear and culture relative to clinical assessment are many. The definition of a “positive test” is debatable—that is, are only double positives counted or are discordant pairs (that by definition contain one positive) counted? The definition affects the outcome of sensitivity and specificity. There would be too many tables to satisfy all readers' curiosities. The purpose of this manuscript was to evaluate either KOH smear or culture relative to a gold standard. For the purposes of rigorous FDA studies, positive disease is defined as a positive KOH smear and culture and clinical assessment; negative disease is defined as a negative KOH smear and negative culture and negative clinical assessment.

### 2.2. KOH Smear and Culture Assay

After cleaning with isopropanol wipes, scrapings were taken with a sterile 15-blade from areas of the foot with the most clinically apparent disease. These areas were typically interdigital and scaling. Harvested scale was smeared onto a slide, and a drop of KOH 10% solution was then added, followed by coverslip and gentle heating with the flame from a match. Under 40 x, the specimen was observed for hyphae, arthroconidia, and yeasts within 3 hours of preparation. Sabouraud dextrose agar, Littman-Oxgall agar, and Mycosel were inoculated via sterile technique with the remainder of the scale on the blade after the KOH smear was prepared. The media were incubated at 25°C and room humidity. Cultures were read at weeks 1, 2, and 3. For cultures with growth, a lactophenol-cotton blue preparation was made. Fungi were identified on macroscopic (i.e., color, texture, rate of growth, pigment production) and microscopic (i.e., microconidia, macroconidia, hyphal elements) morphology.

### 2.3. Sensitivity, Specificity, Positive Predictive Value (PPV), and Negative Predictive Value (NPV)

Using clinical assessment as a gold standard, sensitivity and specificity were determined for KOH smear and culture for each component study and for the overall combined data. Prior to calculating overall sensitivity and specificity, a chi-square test for heterogeneity was performed to determine if it was legitimate to pool data from the individual studies [7]. Sensitivity was defined as the proportion of clinically negative assessments that screened negative for KOH smear or culture. Specificity was defined as the proportion of clinical failures that screened positive for KOH smear or culture. Each sensitivity estimate was accompanied by exact 95% binomial confidence limits.

Prior to calculating KOH smear and culture sensitivity, specificity, PPV, and NPV, sensitivity and specificity generating cross-tabs from the component studies Tables 5(b)–5(f) were subjected to chi-square tests for heterogeneity with *k*-1 degrees of freedom (where *k* = number of component studies). If the chi-square heterogeneity statistics (*χ* ^2^_*α*,k−1_) were not statistically significant (*P* > .05), it would be legitimate to pool the component study data. The *χ* ^2^_0.05,4_ value for culture sensitivity, specificity, PPV, NPV, and spontaneous cure rate was 0.233 (*P* = .97) while that for KOH smear parameters was 7.427 (*P* = .11). It was thus permissible to calculate sensitivity and specificity for the pooled study data.

### 2.4. Data Analysis

All statistical procedures were performed as two-tailed tests using the SAS statistical package, Version 8.2. Differences were considered statistically significant if *P* < .05.

## 3. Results

### 3.1. Study Population

The 484 vehicle patients in this pooled analysis represented 26% of the patient population of the combined studies (*N* = 1,884). There were no major differences with regard to demographic variables between the patients randomized to active treatment or vehicle in any of the component studies. The age of the study patients ranged from 18 to 83 years with a mean of 38.6 ± 13.1 (SD) years (Table 1). The race proportions were 68% Caucasian, 10% Black, 20% Hispanic, and 2% other (Table 2). Males (*N* = 368) comprised 76% of this population, while females (*N* = 116) comprised the other 24% (Table 3). The demographic breakdowns within the component studies were for the most part consistent.

### 3.2. Sensitivity and Specificity

Twenty-four patients were eliminated from evaluation due to incomplete follow-up. Among the 460 patients with complete data, 294 (64%) had a negative KOH smear at 6 weeks, 139 (30%) had negative culture results, and the number with clinically negative exams was 187 (41%) (Table 4).

Using clinical assessment as the gold standard, the sensitivities for KOH smear and culture were 73.3% (95% CI: 66.3 to 79.5%) and 41.7% (34.6 to 49.1%), respectively (Table 5). The difference between the KOH smear and culture sensitivities was statistically significant (*P* < .0001, 2-tailed Fishers exact test). The culture sensitivities of the component studies were confined to a relatively narrow range of 39.1 to 46.3% while those for the KOH smear varied more widely from 58.7 to 91.3%. The highest individual culture sensitivity, 46.3%, was lower than the lowest KOH smear sensitivity of 58.7%. The respective specificities for culture and KOH smear were 77.7% (72.2% to 82.5%) and 42.5% (36.6% to 48.6%). The difference between the culture and KOH smear specificities was also statistically significant (*P* < .0001). The culture specificities of the component studies were again confined to a narrow range of 71.1% to 83.3% while those for the KOH smear varied more widely from 20.5% to 68.7%. The highest individual KOH smear specificity, 68.7%, was lower than the lowest culture specificity of 71.1%. 

### 3.3. Positive and Negative Predictive Value

Table 5 shows the positive predictive value (PPV) and negative predictive value (NPV) calculations for the overall combined population with complete data (*N* = 460). The PPV of culture was 66.0% (95% CI: 60.6%–71.2%) and the NPV of culture was 56.1% (95% CI: 47.4%–64.5%). The PPV of KOH smear was 69.9% (95% CI: 62.3%–76.7%) and the NPV of KOH smear was 46.6% (95% CI: 40.8%–52.5%). The difference between the KOH smear and culture NPV was not statistically significant (*P* = .08, 2-tailed Fishers exact test). The difference between the KOH smear and culture PPV was not statistically significant (*P* = .42, 2-tailed Fishers exact test).

## 4. Discussion

There are 4 major subtypes of tinea pedis: interdigital, moccasin, ulcerative, and inflammatory. Depending on the particular subtype of disease with which a patient presents, a clinician is faced with a subset of differential diagnoses to consider, including allergic contact dermatitis, dyshidrotic eczema, candidiasis, psoriasis, erythrasma, and keratoderma. Likely as a result of the broad scope of this differential, several studies have found that clinical suspicion alone is often insufficient to diagnose tinea pedis [3]. Indeed, conventional dermatology espouses that positive fungal culture is necessary for definitive diagnosis [3–5].

Our study examines the sensitivity and specificity of both KOH smear and fungal culture in determining the presence or absence of tinea pedis, using clinical assessment as the gold standard. While useful as *a* gold standard to compare sensitivity and specificity of KOH smear and culture, clinical disease alone cannot be regarded as a perfect gold standard because a wide variety of nondermatophyte conditions cause identical clinical symptoms. In this study, entrance criteria required positive culture, KOH, and clinical assessment. Creams used were placebo. Therefore, any clinically positive subject at week 4 or 6 would likely be positive because of tinea rather than other conditions. Using KOH examination or fungal culture as gold standards for diagnosis is also problematic. It is possible for culture to be negative even in the presence of active disease. This is supported by the fact that 59 of the 460 patients enrolled in this study had negative cultures at day 28 of the study, but positive cultures at day 42. It is likely that a large proportion of these patients had false negative cultures at day 28. The data from our studies also indicate that, in 185 out of 460 (40.2%) patients, KOH smear and fungal culture results did not correlate at day 42 of the study. Finally, one could consider using clinical positive AND KOH positive as the gold standard for culture, and clinical positive AND culture positive as the gold standard for KOH. In this way, the gold standard might reflect the actual presence or absence of disease better than clinical assessment alone.

The perfect gold standard for infection must be a triple confirmation: positive KOH smear, positive culture, and a clinical exam consistent with tinea pedis. The results of our pooled analysis show KOH smear and fungal culture to be complementary laboratory exams, with higher sensitivity in the former test, and higher specificity in the latter.

One strength of our study was that the pooled analysis was done on data from 5 studies that shared amongst them one clinical investigator and one laboratory performing KOH smears and fungal cultures. The consistency of the results amongst each of the 5 studies (Table 4) confirms the validity of our results. Having all 5 studies completed at the same investigative center is also a potential weakness of the study as it becomes harder to extrapolate the results to other clinical settings. This is particularly true for the NPV and PPV, which are both related to disease prevalence in a particular population. It is unclear if the NPV and PPV can be extrapolated to other regions without knowing their local disease prevalence rates. Additionally, sensitivity and specificity are only as good as the person performing the exam. Our findings were reached at the hands of skilled laboratory technicians. Less experienced observers would be expected to have lower sensitivity and specificity rates for their tests.

KOH smear serves as a good screening test for determining presence of disease, both before and at the end of therapy. A fungal culture, which can take up to three weeks to become positive, can serve as a more specific confirmatory test. The costs of empiric treatment without either laboratory test include the risk of missing an alternate diagnosis and the cost of medication used to treat a nonexistent tinea infection. Still, many suspected cases of tinea pedis are treated empirically, either by medications prescribed by physicians or by patients self-treating with over-the-counter medications. Given the imperfect sensitivity of KOH, this strategy is not completely invalid; however, those who place a high value on KOH may still contend, and justifiably so that an alternate diagnosis can be arrived at more quickly in the presence of a negative KOH.

##  Conflicts of Interest

The authors have no conflicts of interest to disclose insofar as they do not stand to gain anything material from the publication of this manuscript, which was undertaken as an academic pursuit. However, Barrie Levitt, MD is Chief Executive Officer of Taro Pharmaceuticals U.S.A. (Taro USA), Inc. and a major shareholder of its parent company, Taro Pharmaceutical Industries, Ltd. (Taro). Jacob Levitt, MD is a vice president of Taro USA and a major shareholder of Taro. Howard Yanofsky, MD was contracted by Taro USA to perform the studies. Arash Akhavan, MD has no conflicts to declare.

## Figures and Tables

**Table 1:** Age in years by study.

Study	*N*	Mean	Standard Deviation	Minimum	Maximum
STUDY #1	100	37.1	12.2	18	70
STUDY #2	87	38.6	14.0	19	83
STUDY #3	87	39.0	12.8	19	71
STUDY #4	89	39.5	13.4	18	81
STUDY #5	121	39.2	13.1	18	76

Overall	484	38.6	13.1	18	83

**Table 2:** Race distribution by study.

Study	Caucasian		African		Hispanic		Other	
	*N*	%	*N*	%	*N*	%	*N*	%
STUDY #1	61	61.0	14	14.0	24	24.0	1	1.0
STUDY #2	73	83.9	6	6.9	7	8.1	1	1.2
STUDY #3	52	59.8	13	14.9	19	21.8	3	3.5
STUDY #4	58	65.2	10	11.2	19	21.4	2	2.3
STUDY #5	87	71.9	6	5.0	27	22.3	1	0.9

Overall	331	68.4	49	10.1	96	19.8	8	1.7

**Table 3:** Sex distribution by study.

Study	Male		Female	
	*N*	%	*N*	%
STUDY #1	69	69.0	31	31.0
STUDY #2	67	77.0	20	23.0
STUDY #3	70	80.5	17	19.5
STUDY #4	70	78.7	19	21.4
STUDY #5	92	76.0	29	24.0

Overall	368	76.0	116	24.0

**Table 4:** Cure rates at six weeks by study.

Study	N	KOH Negative		Culture Negative		Mycologic Cure*		Clinical Cure	
		*N*	%	*N*	%	*N*	%	*N*	%
STUDY #1	99	81	82	27	27	24	24	45	45
STUDY #2	85	64	75	31	36	29	34	41	48
STUDY #3	81	50	62	19	23	19	23	18	22
STUDY #4	82	51	62	28	34	24	29	37	45
STUDY #5	113	48	43	34	30	26	23	46	41

Overall	460	294	64	139	30	122	27	187	41

*Mycologic cure is defined as negative KOH smear *and* negative culture.

**Table:** (a) Overall (*N* = 460).

		Clinical Cure	
		Yes	No
Culture	Neg	78	61
	Pos	109	212
	Total	187	273
SENSITIVITY (%)		41.7 (34.6–49.1)**		
SPECIFICITY (%)		77.7 (72.2–82.5)		
PV Neg (%)*		56.1 (47.4–64.5)		
PV Pos (%)		66.0 (60.6–71.2)		

	Neg	137	157
KOH Smear	Pos	50	116
	Total	187	273
SENSITIVITY (%)		73.3 (66.3–79.5)		
SPECIFICITY (%)		42.5 (36.6–48.6)		
PV Neg (%)		46.6 (40.8–52.5)		
PV Pos (%)		69.9 (62.3–76.7)		

*PV = Predictive value, Neg = Negative, Pos = Positive

**Point  estimate with exact 95% binomial confidence limits.

**Table:** (b) Study STUDY #1 (*N* = 100).

		Clinical Cure	
		Yes	No
Culture	Neg	18	9
	Pos	28	45
	Total	46	54
SENSITIVITY (%)		39.1 (25.1–54.6)		
SPECIFICITY (%)		83.3 (70.7–92.1)		
PV Neg (%)		66.7 (46.0–83.5)		
PV Pos (%)		61.6 (49.5–72.8)		

KOH Smear	Neg	42	40
	Pos	4	14
	Total	46	54
SENSITIVITY (%)		91.3 (79.2–97.6)		
SPECIFICITY (%)		25.9 (15.0–39.7)		
PV Neg (%)		51.2 (39.9–62.4)		
PV Pos (%)		77.8 (52.4–93.6)		

**Table:** (c) Study STUDY #2 (*N* = 81).

		Clinical Cure	
		Yes	No
Culture	Neg	19	12
	Pos	22	32
	Total	41	44
SENSITIVITY (%)			46.3 (30.7–62.6)
SPECIFICITY (%)			72.7 (57.2–85.0)
PV Neg (%)			61.3 (42.2–78.2)
PV Pos (%)			59.3 (45.0–72.4)
	Neg	29	35
KOH Smear	Pos	12	9
	Total	41	44
SENSITIVITY (%)		70.7 (54.5–83.9)		
SPECIFICITY (%)		20.5 (9.8–35.3)		
PV Neg (%)		45.3 (32.8–58.3)		
PV Pos (%)		42.9 (21.8–66.0)		

**Table:** (d) Study STUDY #3 (*N* = 81).

		Clinical Cure	
		Yes	No
Culture	Neg	8	11
	Pos	10	52
	Total	18	63
SENSITIVITY (%)		44.4 (21.5–69.2)		
SPECIFICITY (%)		82.5 (70.9–90.9)		
PV Neg (%)		42.1 (20.3–66.5)		
PV Pos (%)		83.9 (72.3–92.0)		

KOH Smear	Neg	15	35
	Pos	3	28
	Total	18	63
SENSITIVITY (%)		83.3 (58.6–96.4)		
SPECIFICITY (%)		44.4 (31.9–57.5)		
PV Neg (%)		30.0 (17.9–44.6)		
PV Pos (%)		90.3 (74.2–98.0)		

**Table:** (e) Study STUDY #4 (*N* = 82).

		Clinical Cure	
		Yes	No
Culture	Neg	15	13
	Pos	22	32
	Total	37	45
SENSITIVITY (%)		40.5 (24.8–57.9)		
SPECIFICITY (%)		71.1 (55.7–83.6)		
PV Neg (%)		53.6 (33.9–72.5)		
PV Pos (%)		59.3 (45.0–72.4)		

KOH Smear	Neg	25	26
	Pos	12	19
	Total	37	45
SENSITIVITY (%)		67.6 (50.2–82.0)		
SPECIFICITY (%)		42.2 (27.7–57.9)		
PV Neg (%)		49.0 (34.8–63.4)		
PV Pos (%)		61.3 (42.2–78.2)		

**Table:** (f) Study STUDY #5 (*N* = 113).

		Clinical Cure	
		Yes	No
Culture	Neg	18	16
	Pos	28	51
	Total	46	67
SENSITIVITY (%)		39.1 (25.1–54.6)		
SPECIFICITY (%)		76.1 (64.1–85.7)		
PV Neg (%)		52.9 (35.1–70.2)		
PV Pos (%)		64.6 (53.0–75.0)		

KOH Smear	Neg	27	21
	Pos	19	46
	Total	46	67
SENSITIVITY (%)		58.7 (43.2–73.0)		
SPECIFICITY (%)		68.7 (56.2–79.4)		
PV Neg (%)		56.3 (41.2–70.5)		
PV Pos (%)		70.8 (58.2–81.4)		

## Abbreviations and Acronyms

KOH: Potassium hydroxide
NPV: Negative predictive value
PPV: Positive predictive value
CI: Confidence interval
DTM: Dermatophyte-Test-Medium
SD: Standard deviation
Neg: Negative
Pos: Positive.
